# Diagnostic Contribution of Histopathology in Medicolegal Autopsy: A Denominator-Specific Systematic Review

**DOI:** 10.3390/diagnostics16203308

**Published:** 2026-10-12

**Authors:** Mohammed Sait, Fawaz Almutairi

**Affiliations:** 1 Department of Basic Medical Sciences, College of Medicine, Taibah University, Madinah 42353, Saudi Arabia; 2 Department of Pathology, Faculty of Medicine, King Abdulaziz University, Jeddah 21589, Saudi Arabia; fmnalmutairi@kau.edu.sa

**Keywords:** forensic histopathology, medicolegal autopsy, cause of death, diagnostic yield, systematic review, postmortem examination

## Abstract

**Background/Objectives:** Published estimates of histopathological examination (HPE) contribution in medicolegal autopsy use different denominators and outcome definitions. We examined what these estimates measure and their implications for practice. **Methods:** PubMed, Embase, Web of Science, and Scopus were searched from inception to 31 May 2026 (PROSPERO CRD420261401909). Eligible studies reported denominator-specific HPE contribution. Cause of death (COD) provision and change were distinguished from manner change, confirmation, refinement, and targeted forensic interpretation. JBI checklists supported item-level appraisal. Findings were synthesized narratively without pooling. **Results:** Twenty-one studies were included from 2287 identified records. Strict COD change was reported in 0/100 uncomplicated hanging suicides and 1/189 gross-clear medicolegal autopsies. A broad systematic-histology cohort reported histology-only COD provision in 36/428 cases. In separate unresolved-COD populations, studies reported COD provision in 49/203 and 40/71 cases. These results address different diagnostic questions and do not establish a relationship between uncertainty and yield. Selected denominators, composite outcomes, and incomplete separation from other investigations limited interpretation. Confirmation, injury assessment, and organ-specific findings represented additional functions. **Conclusions:** There is no single transferable HPE yield: the pre-HPE diagnosis, denominator, outcome definition, and contribution of other investigations must accompany each estimate. The review supports clearer reporting and interpretation of HPE contribution; low rates of COD change do not establish when histology can safely be omitted.

## 1. Introduction

Medicolegal autopsy is performed to answer death-investigation questions that cannot be resolved from the circumstances alone. It provides the anatomical basis for determining the cause of death (COD), assessing mechanism and manner where possible, documenting injury and natural disease, and supporting the legal and public-health conclusions that follow [1]. Histopathological examination (HPE) is part of that work, but it is not a substitute for the external and internal autopsy. It is a correlating test. Professional standards recommend histology when no reasonable explanation for the COD is identified after scene and circumstance evaluation, forensic autopsy, toxicology, and, when available, vitreous-fluid analysis, except in skeletonized or severely decomposed remains [1]. Forensic histopathology also has broader interpretive value in assessing disease, injury, and vital reactions that may not be fully resolved macroscopically [2].

The place of HPE in medicolegal autopsy practice remains unsettled. Some departments use broad microscopic sampling to document disease, corroborate gross diagnoses, and preserve a microscopic record, whereas others use histology more selectively, particularly when gross findings already provide a clear explanation for death [2,3,4]. This disagreement has practical implications for workload, reporting time, tissue retention, resource use, and the defensibility of the final opinion [2,3]. Local practice—including jurisdictional rules, staffing, caseload, access to ancillary testing, and tissue-retention policy—can influence when histology is taken [2,4]. Accordingly, a single global baseline cannot be assumed. A broad forensic series with systematic organ histology also reported microscopic contributions to COD provision, injury documentation, and information about prior disease [5].

Before the diagnostic value of HPE can be interpreted, the question being measured has to be clear. A percentage described as “histology contribution” may refer to very different things: provision of a previously unknown COD, change to a provisional COD, confirmation of a suspected diagnosis, refinement of organ pathology, injury characterization, or documentation of additional findings. The denominator is equally important. An estimate based on all medicolegal autopsies is not measuring the same quantity as an estimate based only on cases selected for microscopy. These denominators are not interchangeable. Because denominators and outcome labels have been used inconsistently, the literature contains many study-specific estimates of HPE “utility” but no directly comparable basis for judging when histology changes the medicolegal conclusion. Without preserving both the denominator and the outcome function, a pooled estimate risks combining unlike questions into a single misleading value.

Interpretation should begin with what was known after gross autopsy. When a plausible COD has been recorded, the relevant outcome may be whether microscopy changes, confirms, or refines it. When no COD has been established, provision of a diagnosis becomes a different outcome. These settings differ in diagnostic opportunity by definition; ordering studies by that opportunity cannot independently demonstrate a relationship between uncertainty and yield. Source descriptions of the pre-HPE diagnostic state are nevertheless useful for explaining what each study measured. Defined contexts such as sudden cardiac death also require attention to the specific pathological question and the applicable sampling guidance [6,7].

Previous reviews have examined the broader value of postmortem histopathology. Roulson et al. synthesized clinical-autopsy diagnostic discrepancies and the contribution of histology [8], while Parai and Milroy reviewed the scope and utility of forensic histopathology [2]. More recently, Tortosa López discussed the clinical and legal foundations of histopathology in medicolegal practice [9]. The present review addresses a complementary methodological question: how the outcome-specific denominator, diagnostic state before HPE, and definition of contribution affect interpretation of published estimates. Its objective is to distinguish the functions of HPE reported in medicolegal autopsy studies and identify the information needed for meaningful comparison.

## 2. Materials and Methods

### 2.1. Protocol Registration and Reporting

This systematic review was prospectively registered in PROSPERO (CRD420261401909) and is reported with reference to the PRISMA 2020 guideline [10]; the completed checklist is provided in the Supplementary Materials. Registration was published on 21 May 2026, before formal searching, screening, extraction, and appraisal were recorded as started. The registration states that the uploaded full protocol will be made available after review completion. Structured synthesis without meta-analysis by denominator, sampling strategy, baseline comparator, and ascertainment method was prespecified because substantial heterogeneity was anticipated [11].

During manuscript preparation and revision, ChatGPT (GPT-5.6 Sol; OpenAI) assisted with English-language editing, manuscript organization, consistency checking, and checking cited source material. Claude ([model and version]; Anthropic) assisted with placement and formatting of author-created figures in the journal template. Machine-assisted translation supported assessment of the French-language report, as described in Section 2.3. The authors reviewed and edited the AI-assisted output and retained responsibility for review decisions, source verification, interpretation, and final content.

### 2.2. Eligibility Criteria

Eligible studies were original human studies of autopsies conducted for a medicolegal, forensic, coronial, medical-examiner, or judicial purpose, including hospital-based autopsies performed under medicolegal authority, that evaluated HPE of autopsy tissue in relation to death investigation and reported, or allowed extraction of, at least one denominator-specific outcome of HPE contribution. Contribution had to be extractable relative to a stated or reconstructable gross-autopsy, macroscopic, provisional, or pre-HPE baseline. Eligible outcomes included COD provision, COD change or modification, manner or mechanism change, confirmation of gross findings, diagnostic refinement, occult natural disease detection, injury dating or vitality assessment, and non-contributory, inconclusive, or technically limited HPE. Adult and general medicolegal cohorts were eligible. Mixed adult–pediatric cohorts were eligible when they represented general practice rather than a pediatric-specific population, or when adult/non-pediatric results were separately extractable. Sex and age composition were extracted when reported.

Studies were excluded if they were not eligible original studies; fell outside the medicolegal/autopsy setting; did not evaluate HPE as a separable component of death investigation; lacked an extractable HPE-utility outcome or denominator; or reported only clinical-autopsy, imaging, toxicology, microbiology, or molecular findings without separable HPE contribution. Studies designed specifically for pediatric populations, including infants and sudden unexpected deaths in infancy or childhood, were excluded. Case reports, reviews, editorials, letters without original denominators, conference abstracts without extractable diagnostic-contribution data, and lesion-prevalence studies without diagnostic-contribution data were also excluded. Organ-, disease-, mechanism-, and special-context studies were eligible when they addressed a medicolegal autopsy question, but their restricted denominators were preserved in synthesis and not interpreted as routine all-autopsy yield. In otherwise eligible studies, additional outcomes inseparable from ancillary investigations were retained only as context, not as HPE-specific effects.

### 2.3. Information Sources and Study Selection

MEDLINE via PubMed, Embase, Web of Science Core Collection, and Scopus were searched from inception to 31 May 2026, without restrictions by language, date, article type, publication stage, or full-text availability. Reference lists of eligible studies and relevant reviews were screened, and backward and forward citation searching was performed using Google Scholar, Scopus, and Web of Science; no additional eligible studies were identified. Full strategies, database coverage, and record counts are provided in Table S1.

Records were deduplicated in EndNote. Titles, abstracts, and full texts were screened independently by two reviewers, except for Chatelain et al. [12], whose eligibility was assessed by one reviewer and checked by the second using machine-assisted translation. Disagreements were resolved by discussion. Unretrieved reports were counted separately and were not assessed. Retrieved full texts were in English or French.

### 2.4. Data Extraction and Outcome Standardization

Data were independently extracted by two reviewers into a standardized Microsoft Excel form, capturing study year, country, setting, source population, total autopsies, HPE-submitted cases, analyzed denominator, sampling strategy, case selection, pre-HPE diagnostic state, outcomes, numerators and denominators, ancillary-test separability, and extraction caveats. Disagreements were resolved by discussion against the source reports. The second reviewer’s extraction of the French-language report was completed during revision using machine-assisted translation. No unpublished information was sought from investigators.

Extraction was denominator-specific: each percentage used the denominator closest to the reported outcome, including all eligible autopsies, HPE-submitted cases, gross-pending cases, organ-specific cases, mechanism-specific cases, or special-subgroup cases. Denominators were not interchanged across outcomes. Strict outcomes were prioritized as COD provided, provisional COD changed, and manner or mechanism changed. These were kept separate from confirmation, supportive or additive findings, diagnostic refinement, injury dating or vitality assessment, and author-defined composites, such as “useful”, “contributory”, “diagnostic”, “conclusive”, or “provided/modified”. When provision and change could not be separated, the result was retained as a single composite and not double-counted.

### 2.5. Item-Level Quality Appraisal and Interpretive Limitations

Item-level appraisal used the relevant Joanna Briggs Institute (JBI) checklist: the 10-item case-series checklist for most studies [13] and the eight-item analytical cross-sectional checklist [14] for Mukherjee et al. [15]. Both authors independently completed the item-level appraisals and resolved disagreements by discussion against the source reports and checklist criteria. Table S3 presents the responses (yes, no, unclear, or not applicable) and their explanations. No numerical quality scores, author-defined overall concern categories, or appraisal-derived weights were used.

Additional features informing narrative interpretation were described alongside each study: selection of cases for HPE, clarity of the outcome denominator, ascertainment before and after microscopy or retrospectively, separation of diagnostic change from confirmation, and attribution to HPE rather than other investigations.

### 2.6. Narrative Synthesis and Study-Specific Proportions

Findings were organized by the diagnostic setting reported in the source: an apparent COD or fatal mechanism, an interim diagnosis or mixed HPE cohort, and a COD unresolved after gross examination. Organ-specific, mechanism-specific, and other restricted studies were described separately. These groups were developed during the review for descriptive synthesis, not as a validated classification or predictive model. Studies lacking an explicit pre-HPE stratum were retained as mixed or unstratified cohorts. Because COD provision and COD change have different starting states, their proportions were not compared as a test of an uncertainty–yield association.

Source-reported outcome counts and percentages are displayed with their applicable denominators in Table S5. Rounded source percentages were retained without reconstructing exact event counts. Outcomes were neither pooled nor used to rank studies across different settings, and no review-calculated confidence intervals were presented. Unreconciled diagnostic-contribution estimates were excluded from numerical synthesis, while the source categories and discrepancies were documented in Table S5. Alternative definitions applied to the same cohort were reported separately and were not treated as independent samples.

Consistent with the registration, only published studies were sought, and no formal assessments of bias due to missing results or certainty of evidence were undertaken. Statistical assessment of small-study effects was not performed because no common effect estimate was synthesized and too few comparable studies were available within outcome strata. Publication and selective-reporting biases were considered qualitatively in the Discussion, in addition to the registered methods. Missing outcomes and unavailable denominators were documented in the extraction tables.

The registered sensitivity analysis excluding mixed-age or unclear-age cohorts was not performed. We retained the eligible general-practice cohorts in the descriptive synthesis despite their varying age composition, outcome definitions, and denominators; this approach does not test age-related robustness. Inconsistent reporting of age-linked HPE contribution also limited age-stratified comparisons.

As part of the structured narrative synthesis, recurring reporting limitations encountered during extraction and synthesis were summarized, and practical reporting elements for future studies were outlined from these observations.

## 3. Results

### 3.1. Study Selection

**Figure 1:**
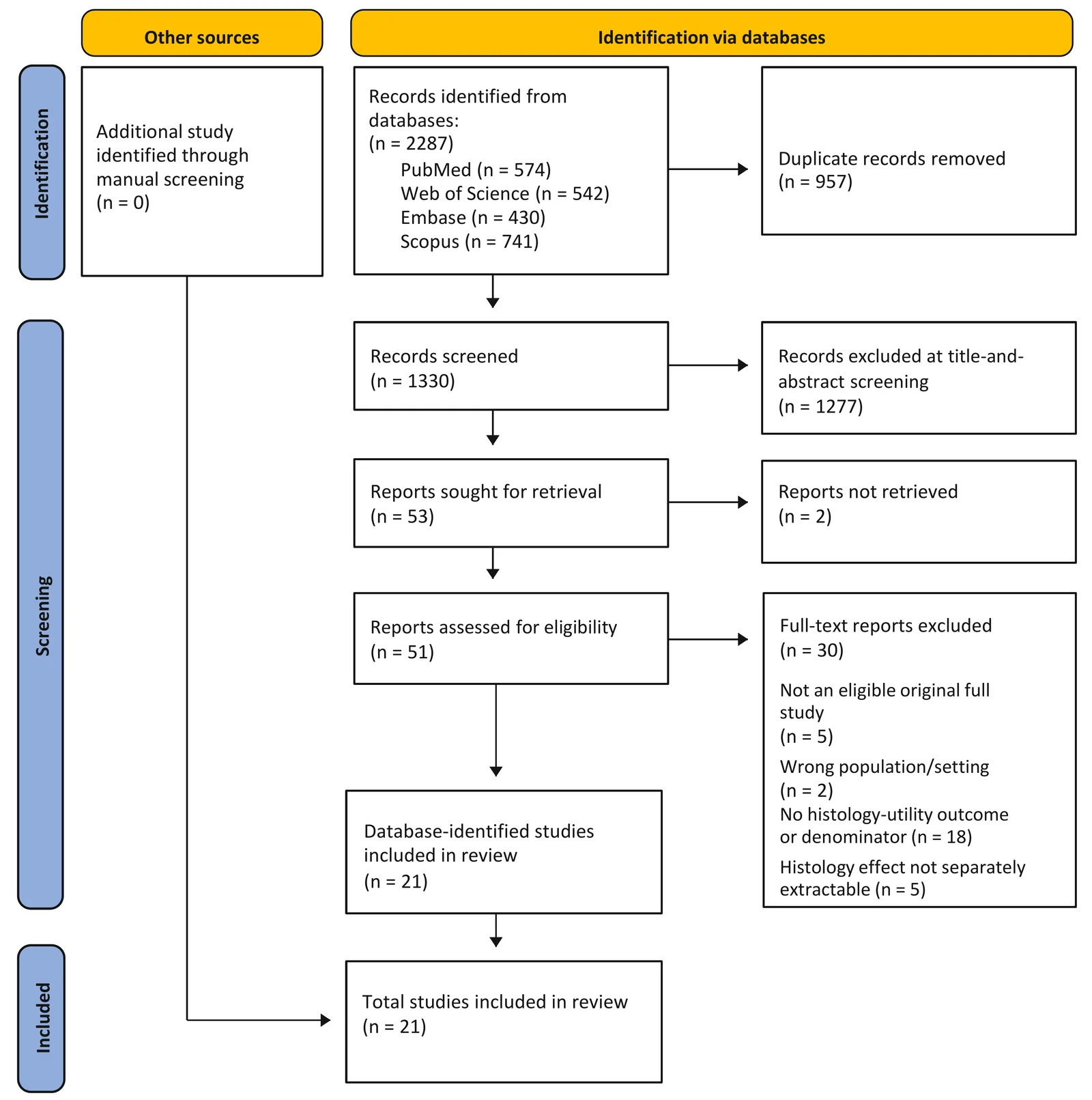
PRISMA 2020 flow diagram of study selection.

The database searches identified 2287 records. After removal of 957 duplicates, 1330 records underwent title-and-abstract screening, of which 1277 were excluded. Fifty-three reports were sought for retrieval; two could not be retrieved, leaving 51 reports assessed for eligibility. Thirty reports were excluded: five were not eligible original studies, two had the wrong population or setting, eighteen lacked an extractable HPE-utility outcome or denominator, and five did not report a separately extractable HPE effect. Twenty-one studies met the inclusion criteria. The study-selection process is shown in Figure 1. Reports excluded after eligibility assessment are listed with reasons in Table S2, which also identifies the two reports not retrieved.

### 3.2. Included Studies and Interpretability

The 21 studies covered broad forensic or coronial practice, selected HPE submissions, gross-clear cases, and restricted organ, disease, mechanism, exhumation, alcohol-related, or retention-policy settings. Table 1 identifies each study’s source population, HPE denominator, and principal interpretive limitation. Table 2 illustrates distinct diagnostic questions, placing the outcome definition and relevant denominator beside each finding. It is not an exhaustive comparison or a ranking of studies; detailed outcomes from every included study, including contextual studies, are provided in Table S5.

Among the 20 studies assessed with the case-series checklist, standardized and reliable measurement was unclear in 11 (Q2). Source-specific concerns included unclear outcome reporting and statistical analysis in Das et al. (Q8 and Q10). In the analytical cross-sectional appraisal of Mukherjee et al., confounding factors and strategies to address them were not reported (Q5 and Q6). Item-level responses and explanations are provided in Table S3.

### 3.3. Cases with an Apparent COD or Fatal Mechanism

In studies designed around an apparent diagnosis or uncomplicated mechanism, strict changes were infrequent within the sampled populations. Tse et al. reported no change in COD, mechanism, or manner in 100 uncomplicated hanging suicides [32]. Molina et al. reported COD modification in 1/189 gross-clear medicolegal autopsies and no manner change; gross-uncertain cases and cases submitted for injury dating or disease characterization had been excluded [26]. These selection features are essential to interpreting the findings.

Beer et al. studied 200 selected trauma-related deaths. Both reviewing pathologists attributed a COD change to HPE in 1/200 cases; at least one reviewer did so in 8/200. The single both-reviewer COD change also involved a manner change, so these are not two independent events. Certainty increased in 17/192 cases without an either-reviewer COD change [4].

### 3.4. Interim Diagnoses and Mixed or Selected HPE Cohorts

Where an interim COD had been recorded, Langlois reported modification in 21/435 cases and confirmation in 158/435 [24]. Other studies did not supply a comparable pre-HPE stratum. Fronczek et al. reported histology-alone COD provision in 5/287 HPE cases from a source population of 500 autopsies [20]. Chatelain et al. reported 35/400 microscopic major diagnoses related to death, distinct from 48 diagnoses identified by additional gross examination; 213/400 examinations confirmed gross findings or assisted injury dating [12]. These broad cohorts were not classified as gross-clear cases. In de la Grandmaison et al. [5], 428 randomly selected cases were reviewed from an institutional series of 1786 forensic autopsies with systematic organ histology. HPE alone established COD in 36/428 (8.4%); the analyzed cohort was not restricted to gross-clear cases and no separate pre-HPE unresolved denominator was reported [5]. The population was a general forensic cohort (age range 5–91 years), with sudden infant death syndrome cases excluded.

Among more recently selected cohorts, Bansal et al. distinguished between 3/54 cases in which HPE moved the COD from an undetermined to a cardiac category and 4/54 cases involving changes within existing categories [17]. Shubhendu et al.’s reported 33/172 definitive CODs, whether provided or modified, cannot be divided into those two functions [30]. Mukherjee et al.’s final opinions based on HPE are contextual composite outcomes: the reference opinion itself incorporated HPE, preventing interpretation as independently assessed diagnostic change [15].

### 3.5. COD Unresolved After Gross Examination

In source-defined unresolved strata, Langlois reported COD provision in 49/203 pending cases [24], Ambade et al. in 12/64 cases without an apparent COD [16], and Matkowski and Benbow in 40/71 gross-unascertained cases [25]. The latter study also reported 24 altered interim diagnoses within its wider HPE cohort. These are COD-provision findings within unresolved-case denominators, not all-autopsy yield estimates.

### 3.6. Targeted Interpretation and Combined Investigations

Mechanism- and organ-specific studies addressed additional forensic questions. Perju-Dumbravă et al. examined neck tissues in 16 of 66 hanging autopsies. The report describes microscopic confirmation of a suspicion of manual strangulation that was already raised by gross findings. This is targeted corroborative evidence, not an independently demonstrated new manner-of-death diagnosis or a general HPE-yield estimate [27]. Chaganti et al. similarly addressed ligature-site vitality and a separate organ-HPE subgroup [18]. Cardiac, brain, exhumation, and alcohol-related cohorts are retained within their restricted denominators [22,23,29,31].

Das et al. reported complete autolysis in 2/28 selected cases, an extractable, technically limited HPE outcome eligible under Section 2.2. The remaining 26 examinations comprised 14 obscure-COD and 12 diseased-organ cases. Conflicting diagnostic-contribution labels and counts could not be reconciled; those estimates were excluded from numerical synthesis, with the source details retained in Table S5 [19]. In exhumation and alcohol-related studies, findings involving toxicology or microbiology are described as combined contributions rather than effects attributable to HPE alone [23,31].

De la Grandmaison et al. also reported mechanisms not demonstrated grossly in 173/428 cases, additional information on prior medical conditions in 211/428, and better injury documentation in 94/428 [5]. These outcomes can overlap. Microscopic findings reportedly affected manner in 56/428 cases, but toxicology contributed to excluding suspected overdose in one case; the whole manner count therefore cannot be treated as an isolated HPE effect. Further outcomes and source caveats are retained in Table S5.

**Table 2:** Selected denominator-specific findings arranged by the diagnostic setting before HPE.

Study	Denominator and Diagnostic Setting	Reported Contribution	Interpretation and Attribution
Tse et al. (2012) [32]	100 uncomplicated hanging suicides	COD, mechanism, or manner changes: 0/100.	Restricted uncomplicated-hanging comparator.
Molina et al. (2007) [26]	189 gross-clear autopsies	COD modified: 1/189. Manner changed: 0/189.	Gross-uncertain and other targeted cases excluded.
Beer et al. (2023) [4]	200 selected trauma deaths	COD changed: 1/200 by both reviewers; 8/200 by either reviewer.	Both-reviewer manner change was the same single case. Definitions are alternative readings of one cohort.
Langlois (2006) [24]	435 interim-COD cases within 638 autopsies	Interim COD modified: 21/435; confirmed: 158/435.	Modification and confirmation are separate outcomes.
de la Grandmaison et al. (2010) [5]	428 random cases from 1786 autopsies; systematic HPE	Histology-alone COD provision: 36/428 (8.4%).	Broad cohort; no separate pre-HPE unresolved denominator. This is provision, not strict COD change.
Fronczek et al. (2014) [20]	287 HPE cases from 500 autopsies	Histology-alone COD provision: 5/287.	No separately defined gross-clear stratum.
Chatelain et al. (2012) [12]	400 HPE cases from 536 autopsies	Microscopic major diagnoses related to death: 35/400. Confirmation or injury dating: 213/400.	The broader 83/400 includes 48 diagnoses made by additional gross examination.
Bansal et al. (2025) [17]	54 HPE cases from 171 autopsies	From undetermined to cardiac COD: 3/54; existing COD category changed: 4/54.	HPE effects tabulated separately from toxicology.
Langlois (2006) [24]	203 pending-COD cases within 638 autopsies	COD provided: 49/203.	Pending-case denominator; different outcome from changing an existing COD.
Ambade et al. (2016) [16]	64 not-apparent cases within 263 HPE cases	COD provided: 12/64.	The broader 47/263 includes support/refinement in apparent-COD cases.
Matkowski et al. (2021) [25]	71 gross-unascertained cases within 141 HPE cases	COD provided: 40/71.	The broader 64/141 direct-COD composite also includes 24 altered interim diagnoses.

COD, cause of death; HPE, histopathological examination. Zero denotes an assessed outcome with no events; unreported or inseparable outcomes are not zero. Rows illustrate different diagnostic questions and are not directly comparable or additive. Detailed outcomes and caveats for all 21 studies are in Table S5.

### 3.7. Reporting Limitations

Recurring limitations included incomplete case-flow reporting, unstated sampling strategy, absent pre-HPE diagnosis, composite outcome labels, and incomplete separation of histology from other investigations. Table S4 summarizes the reporting problems and the information needed to resolve them.

## 4. Discussion

### 4.1. Principal Findings

The literature measures several different functions under the label of HPE contribution. Strict COD changes were infrequent in selected gross-clear or uncomplicated-mechanism cohorts. In a broad cohort with systematic histology, de la Grandmaison et al. reported histology-only COD provision in 36/428 cases [5]; this addresses a different outcome in a different population. Studies of unresolved deaths reported COD provision, while other studies measured confirmation, refinement, injury interpretation, or broader diagnostic usefulness. The review’s contribution is to clarify what each estimate measures and the population to which it applies.

### 4.2. Denominators and Diagnostic Opportunity

An HPE proportion is interpretable only when the reader knows what was unresolved before microscopy, how cases were selected, and what counted as a contribution. All-autopsy denominators, selected HPE submissions, gross-pending cases, and organ- or mechanism-specific cohorts answer different questions. Provision of an unresolved COD, alteration of a provisional diagnosis, and confirmation of an existing diagnosis should therefore remain separate. Preserving these distinctions improves interpretation without requiring a common numerical estimate.

The descriptive diagnostic settings are not a validated uncertainty scale. COD provision is possible only where a COD was previously unresolved, whereas COD change requires an existing diagnosis. An unresolved starting state does not guarantee that microscopy will resolve the case. These differing opportunities and endpoints nevertheless prevent interpreting the displayed proportions as a test of whether uncertainty independently predicts yield. Differences may also reflect case selection, sampling intensity, and attribution to other investigations.

### 4.3. Strict COD or Manner Change and Broader Forensic Value Are Different Outcomes

A low rate of strict COD or manner change should not be interpreted as absence of value. In medicolegal practice, confirmation and refinement may be important even when they do not alter the final death certificate. HPE may strengthen the pathological basis of a gross diagnosis, document natural disease or injury, identify competing pathology, assist clinicopathological and circumstantial correlation, and preserve a microscopic record for later review [2,3]. These functions may be particularly relevant in deaths with legal scrutiny, unexpected circumstances, institutional or custodial settings, possible medical intervention issues, family concern, or public-health implications [1,2]. The commentary and authors’ response that followed Beer et al. illustrate how the same low rate of strict COD change can be read differently depending on which function of histology is being valued [33,34].

However, these functions should be reported separately from strict COD provision or COD change. Combining confirmation, refinement, incidental disease, injury characterization, and strict diagnostic change under broad labels—such as “useful”, “contributory”, “diagnostic”, or “conclusive”—can overstate strict diagnostic impact when these outcomes are interpreted as equivalent, and limits comparison between studies (Table 2 and Table S4). A study showing frequent confirmation of gross findings is not equivalent to a study showing frequent provision of a previously unknown COD. Both observations may be valid, but they answer different forensic questions.

### 4.4. Incorporation Bias and Composite Endpoints

A final opinion that incorporates HPE can describe the conclusion reached after investigation, but it cannot independently validate the accuracy of HPE. Using the same microscopic findings both to define the reference opinion and to establish the correctness of HPE introduces incorporation bias [35,36]. A prospectively recorded change between pre-HPE and post-HPE opinions can still describe diagnostic impact; it does not by itself establish diagnostic accuracy or the consequences of withholding microscopy. Mukherjee et al. reported HPE-based final-opinion rates in gross-pending strata [15]. We retain these as contextual final-opinion outcomes because they do not sparately establish adjudicated COD provision or change independently.

A separate problem is outcome composition. Labels such as “provided/modified COD”, “conclusive”, “significant”, “effective”, or “based on HPE” may combine provision, alteration, confirmation, and supportive interpretation (Table S4). An inseparable composite is retained under its source definition and cannot establish the frequency of any individual component. Future studies should record the pre-HPE diagnosis and distinguish these outcome functions explicitly, as summarized in Box 1.

When several investigations contribute to a final opinion, attributing the whole result to histology overstates its isolated contribution. Even an explicitly attributed HPE effect remains the source authors’ assessment; it is not automatically an independently validated causal effect or diagnostic-accuracy estimate.

### 4.5. Scenario-Specific Implications for Medicolegal Practice

Table 3 translates the findings into questions for everyday medicolegal interpretation. It is an interpretive summary, not a validated sampling algorithm or practice guideline. The distinction matters because a study reporting few changed diagnoses has not necessarily evaluated missed pathology, later case review, evidential value, or the consequences of not taking tissue.

The reporting elements in Box 1 can support local service evaluation. The observed percentages do not justify a universal omission rule, reduced minimum sampling, or departure from applicable standards and case-specific professional judgment [1,6,7].

Hanging deaths and suspected IHD illustrate the importance of the question being asked. The no-change finding in uncomplicated hanging [32] does not answer whether neck microscopy assists a disputed vitality or mechanism assessment [18,27]. Proposed histological signs in hanging also require interpretation in their studied setting [37]. Cardiac findings must be interpreted within the population and sampling context of each study [24,25,29]. Relevant sudden-death guidance and the continuing discussion of myocardial sampling further illustrate why adequate sampling cannot be inferred from a low overall COD-change rate [6,7,38,39,40,41].

Whole-organ retention poses a further question: Provenzano et al. examined heart and brain sampling within an organ-retention policy, so its broad utility endpoint should be interpreted within that setting [28]. Recent work on alcohol-related death likewise places microscopic findings within a wider pathological, toxicological, and biochemical investigation [42].

### 4.6. Implications for Future Reporting and Research

The recurring limitations in Table S4 support six reporting elements for future studies (Box 1). Prospective recording of the provisional diagnosis and information available at each stage would distinguish newly observed findings from retrospective attribution. Evaluations of sampling policies would also need a defined comparator, systematic assessment of missed diagnoses and evidential consequences, and explicit criteria for acceptable risk.

Box 1 **Six reporting elements for studies of HPE contribution.**

1. Case flow and sampling: report total eligible autopsies, HPE submissions, analyzed cases, exclusions, and the reason and strategy for sampling.

2. Pre-HPE diagnosis: record the provisional COD or state that it remained unresolved before microscopy; identify other pending investigations.

3. Separate outcomes: distinguish COD provision, COD change, manner change, mechanism interpretation, confirmation, refinement, and vitality or injury-timing contribution.

4. Attribution: state whether the contribution was HPE-specific, combined with other investigations, or not separable; retain inseparable source outcomes as composites.

5. Denominators and missingness: give n/N for each outcome and distinguish no impact from absent, autolyzed, inadequate, or inconclusive tissue.

6. Interpretation limits: state whether the findings apply to all autopsies or only to selected cases, organs, mechanisms, or other restricted settings.

### 4.7. Strengths and Limitations

Strengths include prospective registration, a broad search strategy, preservation of outcome-specific denominators, and separation of diagnostic change from confirmation and targeted interpretation. Structured narrative synthesis was appropriate because populations, sampling strategies, pre-HPE states, and outcome definitions were incompatible with a single pooled estimate [11].

Most studies were observational, often retrospective, and many used selected, restricted, or unclear denominators. Small cohorts limit precision, and the descriptive tables provide no review-calculated confidence intervals. Composite outcomes and incomplete separation from other investigations limit comparison and generalizability. The initial single-reviewer eligibility assessment of the French report, reliance on machine-assisted translation, later completion of its second-reviewer extraction and appraisal, and the unperformed age-composition sensitivity analysis are review-process limitations (Section 2.3, Section 2.4, Section 2.5 and Section 2.6). No formal certainty assessment was undertaken. Two reports could not be retrieved, leaving their eligibility unresolved.

Publication and selective-reporting biases cannot be excluded. Unpublished local audits with no additional contribution and preferential publication of unusual positive findings could overrepresent diagnostically productive examinations. Within studies, omission of non-contributory or technically limited examinations and emphasis on favorable categories could inflate reported yield. These are plausible mechanisms, not demonstrated explanations; their net direction and magnitude cannot be estimated from this heterogeneous literature. The absence of a funnel plot or statistical test is not evidence that reporting bias is absent.

## 5. Conclusions

HPE contribution should be reported with the diagnostic state before microscopy, the relevant denominator, the outcome definition, and the role of other investigations. Provision of an unresolved COD, change to a provisional COD, confirmation, refinement, and targeted forensic interpretation are distinct functions. The available studies cannot support a single transferable yield or establish when microscopy can safely be omitted. Their practical value is to improve documentation and interpretation of HPE contribution and to guide better-designed evaluations of sampling practice.

## Figures and Tables

**Table 1:** Included studies, source populations, and limits on interpretation.

Study	Setting	Analyzed Sample and Source Population	Scope and Interpretive Limitation
Ambade et al. (2016) [16]	India; medicolegal autopsy	263 HPE/2134 total medicolegal autopsies; selective HPE	Selected HPE cohort; unresolved subgroup and supportive findings reported separately.
Bansal et al. (2025) [17]	India; community-related brought-dead autopsies	54 HPE/171 community-related brought-dead autopsies; selective HPE	Selected HPE cohort; HPE and toxicology effects tabulated separately.
Beer et al. (2023) [4]	Sweden; selected trauma-related medicolegal deaths	200 selected deaths with HPE; 50 each from fire, immersion, suspension, and traffic deaths	Selected trauma deaths; both-reviewer and either-reviewer attribution differ.
Chaganti et al. (2021) [18]	India; HPE referral/medicolegal autopsy series	364 HPE referral cases; major hanging/ligature-site component	Referral series; ligature-site and organ-subgroup questions.
Chatelain et al. (2012) [12]	France; forensic autopsy histopathology audit	400 HPE/536 forensic autopsies; high-coverage selective HPE	Selected HPE cohort; microscopic diagnoses distinguished from additional gross findings.
Das et al. (2025) [19]	India; selected natural/obscure medicolegal deaths	26 analyzable HPE cases/28 selected natural-or-obscure HPE cases	Technically limited HPE: 2/28 completely autolyzed cases. Conflicting diagnostic-contribution estimates excluded from numerical synthesis.
de la Grandmaison et al. (2010) [5]	France; general forensic autopsy	428 randomly selected cases/1786 institutional autopsies; systematic organ histology	General mixed-age cohort; no separate gross-clear stratum. Manner attribution partly involves toxicology.
Fronczek et al. (2014) [20]	United Kingdom; adult forensic autopsy	287 HPE/500 adult forensic cases; selective HPE	Broad selected HPE cohort; pre-HPE diagnostic stratum not separately defined.
Goel et al. (2023) [21]	India; selected medicolegal HPE cases	100 selected HPE medicolegal cases; total source autopsy denominator unclear	Selected HPE cases; unclear source denominator and author-defined conclusive outcome.
Hutchins et al. (2012) [22]	United States; brain/CNS forensic autopsy histology	195 Miami-Dade brain-histology cases plus 213 Mayo medicolegal autopsies with complete neuropathology	Brain/CNS-only cohorts with different institutional sampling strategies.
Karger et al. (2004) [23]	Germany; forensic exhumations	155 consecutive forensic exhumations; special delayed-PMI subgroup	Exhumation series; preservation and combined investigations limit attribution.
Langlois (2006) [24]	Australia; adult coronial postmortem examinations	638 adult coronial autopsies; systematic extensive histology	Systematic histology; interim-COD and pending-COD strata distinguished.
Matkowski et al. (2021) [25]	United Kingdom; adult coronial autopsies in teaching-hospital setting	141 HPE/532 adult coronial autopsies; selective HPE	Selective HPE; unresolved provision and interim alteration are distinct outcomes.
Molina et al. (2007) [26]	United States; medicolegal autopsy, gross-clear routine cases	189 routine gross-clear medicolegal autopsies; prospective blinded HPE comparator	Gross-clear comparator; gross-uncertain and other targeted cases excluded.
Mukherjee et al. (2025) [15]	India; tertiary-care medicolegal autopsy setting	87 convenience-sampled medicolegal autopsies with HPE; total source denominator unclear	Convenience sample; HPE incorporated into the final reference opinion.
Perju-Dumbravă et al. (2018) [27]	Romania; hanging deaths/neck soft-tissue histology	16 neck-HPE cases/66 hanging deaths	Neck HPE in 16 of 66 hangings; targeted corroboration, not routine COD yield.
Provenzano et al. (2026) [28]	France; medicolegal institute/organ-retention and sampling-policy study	142 analyzed/225 HPE ordered/1068 forensic autopsies; selective HPE in a heart/brain organ-retention policy context	Heart/brain retention and sampling policy; broad utility endpoint.
Rajakarunanaike et al. (2025) [29]	Sri Lanka; cardiac/IHD medicolegal autopsy review	100 deaths concluded as IHD with cardiac microscopic review; organ-specific IHD cohort	Deaths already concluded as IHD; cardiac/disease-specific interpretation.
Shubhendu et al. (2025) [30]	India; medical-board/custodial-heavy medicolegal autopsies	172 analyzed HPE cases/177 HPE-submitted cases/262 medical-board autopsies	Selected medical-board cohort; provided-or-modified COD is composite.
Thomsen (2000) [31]	Denmark; medicolegal autopsies in alcoholics	73 consecutive medicolegal autopsies in alcoholics; systematic histology, toxicology, and microbiology	Alcohol-related deaths; histology, toxicology, and microbiology integrated.
Tse et al. (2012) [32]	Australia; hanging-suicide medicolegal autopsies	100 randomly selected uncomplicated hanging suicides with routine histology	Uncomplicated hanging suicides; mechanism-specific no-change comparator.

HPE, histopathological examination; COD, cause of death; IHD, ischemic heart disease; CNS, central nervous system. Denominators identify the analyzed sample and the source population when available. Item-level JBI appraisal is in Table S3; detailed outcomes are in Table S5.

**Table 3:** Practical interpretation of the evidence and limits on its use.

Setting	What the Evidence Describes	Practical Reporting Implication	What It Does not Establish
Apparent COD or uncomplicated mechanism	Few strict changes in selected cohorts [4,26,32].	Document whether HPE confirms, refines, or changes the provisional opinion.	A low change frequency does not establish safety of omission.
COD unresolved after gross examination	COD provision reported within unresolved-case denominators [16,24,25].	Record the unresolved question and whether HPE or combined investigations resolved it.	These selected fractions do not estimate all-autopsy yield.
Disputed neck injury or vitality	Selected neck-histology and ligature-site evidence [18,27].	Describe the microscopic finding and its relation to gross and circumstantial evidence.	Small selected series do not validate a universal marker or sampling rule.
Cardiac, brain, or other organ-specific question	Organ- and disease-restricted findings and retention-policy outcomes [22,28,29].	State the organ, tissue sampled, diagnostic question, and relevant specialist guidance.	Organ-specific contribution cannot establish general autopsy yield or universal retention policy.
Exhumation or alcohol-related death	Preservation-dependent findings and combined investigations [23,31].	State tissue adequacy and the respective roles of histology, toxicology, and microbiology.	An integrated final opinion is not an isolated HPE effect.
Service evaluation or sampling policy	Heterogeneous denominators and outcomes across the evidence base.	Use the reporting elements in Box 1 to define the local evaluation.	These studies do not establish the safety, costs, or consequences of an omission policy.

COD, cause of death; HPE, histopathological examination. These interpretation and reporting considerations do not establish the safety of omitting HPE or a universal sampling policy. Study-specific outcomes and attribution limits are provided in Table 2 and Table S5.

## Data Availability

The extracted outcome data supporting the reported synthesis are presented in the article and Supplementary Tables S1–S5. Additional source detail is available in the cited primary publications.

## Supplementary Materials

The following supporting information can be downloaded at https://www.mdpi.com/article/10.3390/diagnostics16203308/s1. Supplementary Materials: PRISMA 2020 checklist; Table S1: Electronic database search strategies and records identified before deduplication; Table S2: Full-text reports excluded after eligibility assessment; Table S3: JBI critical appraisal of included studies: (A) item-level responses and (B) explanations of item-level responses; Table S4: Reporting limitations and proposed reporting elements for studies of HPE contribution; Table S5: Detailed denominator-specific outcomes and source caveats for all included studies.

## Abbreviations

The following abbreviations are used in this manuscript:

CNS	Central nervous system
COD	Cause of death
HPE	Histopathological examination
IHD	Ischemic heart disease
JBI	Joanna Briggs Institute
PMI	Postmortem interval
PRISMA	Preferred Reporting Items for Systematic Reviews and Meta-Analyses
PROSPERO	International Prospective Register of Systematic Reviews
