# Supplementary material for: Diagnostic Contribution of Histopathology in Medicolegal Autopsy: A Denominator-Specific Systematic Review
Source: Diagnostics (Basel). 2026 Oct 12;16(20):3308. doi: 10.3390/diagnostics16203308 (PMC13703071; doi:10.3390/diagnostics16203308)
Supplement: Supplementary file 1 [file diagnostics-16-03308-s001.zip › Supplementary Tables S1-S5 final.pdf]

Supplementary Materials

Tables S1–S5

Diagnostic Contribution of Histopathology in Medicolegal Autopsy: A Denominator-Specific Systematic Review

The PRISMA 2020 checklist is provided separately. This document contains Tables S1–S5; Table S5 provides detailed outcomes and source caveats supporting the shorter main Table 2.

Table S1. Electronic database search strategies and records identified before deduplication.

Database	Search field / platform	Search strategy	Filters / limits	Records identified
**PubMed/MEDLINE**	PubMed; MeSH and title/abstract fields	("Forensic Pathology"[Mesh] OR "forensic autopsy"[tiab] OR "forensic autopsies"[tiab] OR "medico-legal autopsy"[tiab] OR "medico-legal autopsies"[tiab] OR "medicolegal autopsy"[tiab] OR "medicolegal autopsies"[tiab] OR "coronial autopsy"[tiab] OR "coronial autopsies"[tiab] OR "coroner autopsy"[tiab] OR "coroner autopsies"[tiab] OR "medical examiner autopsy"[tiab] OR "medical examiner autopsies"[tiab] OR "forensic postmortem"[tiab] OR "forensic post-mortem"[tiab] OR "forensic necropsy"[tiab] OR "forensic necropsies"[tiab])<br>*AND*<br>(histolog*[tiab] OR histopatholog*[tiab] OR microscop*[tiab])<br>*AND*<br>(utility[tiab] OR usefulness[tiab] OR contribution[tiab] OR yield[tiab] OR value[tiab] OR "diagnostic yield"[tiab] OR "diagnostic value"[tiab] OR "cause of death"[tiab] OR "manner of death"[tiab] OR "gross findings"[tiab] OR "macroscopic findings"[tiab] OR "pre-histology"[tiab] OR "pre histology"[tiab] OR "changed the diagnosis"[tiab] OR "change in diagnosis"[tiab])	None	574
**Scopus**	Scopus; TITLE-ABS-KEY	TITLE-ABS-KEY(("forensic autopsy" OR "forensic autopsies" OR "medico-legal autopsy" OR "medico-legal autopsies" OR "medicolegal autopsy" OR "medicolegal autopsies" OR "coronial autopsy" OR "coronial autopsies" OR "coroner autopsy" OR "coroner autopsies" OR "medical examiner autopsy" OR "medical examiner autopsies" OR "forensic postmortem" OR "forensic post-mortem" OR "forensic necropsy" OR "forensic necropsies")<br>*AND*<br>(histolog* OR histopatholog* OR microscop*)<br>*AND*<br>("diagnostic yield" OR "diagnostic value" OR "diagnostic contribution" OR contribution OR useful* OR utility OR yield OR value OR "cause of death" OR "manner of death" OR "gross findings" OR "macroscopic findings" OR "pre-histology" OR "pre histology" OR "changed the diagnosis" OR "change in diagnosis"))	None	741

Database	Search field / platform	Search strategy	Filters / limits	Records identified
**Web of Science Core Collection**	Web of Science Core Collection; Topic search (TS)	TS=(("forensic autopsy" OR "forensic autopsies" OR "medico-legal autopsy" OR "medico-legal autopsies" OR "medicolegal autopsy" OR "medicolegal autopsies" OR "coronial autopsy" OR "coronial autopsies" OR "coroner autopsy" OR "coroner autopsies" OR "medical examiner autopsy" OR "medical examiner autopsies" OR "forensic postmortem" OR "forensic post-mortem" OR "forensic necropsy" OR "forensic necropsies" OR "forensic pathology")<br>*AND*<br>(histolog* OR histopatholog* OR microscop*)<br>*AND*<br>(utility OR usefulness OR contribution OR yield OR value OR "diagnostic yield" OR "diagnostic value" OR "cause of death" OR "manner of death" OR "gross findings" OR "macroscopic findings" OR "pre-histology" OR "pre histology" OR "changed the diagnosis" OR "change in diagnosis"))	None	542
**Embase**	Embase; title/abstract/keyword fields	('forensic autopsy':ti,ab,kw OR 'forensic autopsies':ti,ab,kw OR 'medico-legal autopsy':ti,ab,kw OR 'medico-legal autopsies':ti,ab,kw OR 'medicolegal autopsy':ti,ab,kw OR 'medicolegal autopsies':ti,ab,kw OR 'coronial autopsy':ti,ab,kw OR 'coronial autopsies':ti,ab,kw OR 'coroner autopsy':ti,ab,kw OR 'coroner autopsies':ti,ab,kw OR 'medical examiner autopsy':ti,ab,kw OR 'medical examiner autopsies':ti,ab,kw OR 'forensic postmortem':ti,ab,kw OR 'forensic post-mortem':ti,ab,kw OR 'forensic necropsy':ti,ab,kw OR 'forensic necropsies':ti,ab,kw)<br>*AND*<br>(histolog*:ti,ab,kw OR histopatholog*:ti,ab,kw OR microscop*:ti,ab,kw)<br>*AND*<br>(utility:ti,ab,kw OR usefulness:ti,ab,kw OR contribution:ti,ab,kw OR yield:ti,ab,kw OR value:ti,ab,kw OR 'diagnostic yield':ti,ab,kw OR 'diagnostic value':ti,ab,kw OR 'diagnostic contribution':ti,ab,kw OR 'cause of death':ti,ab,kw OR 'manner of death':ti,ab,kw OR 'gross findings':ti,ab,kw OR 'macroscopic findings':ti,ab,kw OR 'pre-histology':ti,ab,kw OR 'pre histology':ti,ab,kw OR 'changed the diagnosis':ti,ab,kw OR 'change in diagnosis':ti,ab,kw)	None	430
**Total before deduplication**	—	—	—	**2287**

Note. *Searches were run without date, language, article-type, publication-stage, or full-text availability filters. Counts are pre-deduplication database yields. The Scopus and Embase strategies reflect the refined searches used for the updated counts. TS, Topic search; TITLE-ABS-KEY, title, abstract, and keyword fields; ti,ab,kw, title, abstract, and keyword fields.*

Table S2. Full-text reports excluded after eligibility assessment.

This table lists the 30 reports excluded after eligibility assessment. The two reports sought but not retrieved are identified in the note; neither is counted as an assessed exclusion.

Authors, year	Title	Primary exclusion reason
**Aquila et al. (2018)**	Electric or traumatic injury? The role of histopathological investigations	Category 1 - two-case report/very small case series; no systematic denominator.
**Hamilton et al. (2015)**	The Importance of Microscopic Examination of the Lungs in Decedents with Sustained Central Intravascular Catheters: A Nine-Case Series	Category 1 - selected nine-case series; no systematic eligible autopsy denominator.
**Parai and Milroy (2018)**	The Utility and Scope of Forensic Histopathology	Category 1 - invited narrative review/background article; not a primary study.
**Savall et al. (2014)**	Acute Aortic Dissection Diagnosed after Embalming: Macroscopic and Microscopic Findings	Category 1 - single case report; no systematic denominator or utility-yield analysis.
**Suess and Hausmann (2020)**	Gross and Histopathological Pulmonary Findings in a COVID-19 Associated Death During Self-Isolation	Category 1 - single forensic COVID-19 autopsy case report.
**Wu et al. (2015)**	Evaluation of the Cardiac Conduction System in a Blended (Medical-Forensic) Autopsy Practice: A Review of 215 Cases (1994–2013)	Category 2 – mixed medical/forensic autopsy cohort; the abstract does not separately identify a legally authorized medicolegal subset and its contribution outcomes. Conference-abstract status alone is not the reason for exclusion.
**Latten et al. (2019)**	The Needle in the Haystack: Histology of Post-Mortem Computed Tomography Guided Biopsies Versus Autopsy Derived Tissue	Category 2 - clinical/internal-medicine postmortem biopsy setting; outside the eligible medicolegal autopsy setting.
**Amadasi et al. (2016)**	Macroscopic and Histopathological Aspects of Chemical Damage to Human Tissues Depending on the Survival Time	Category 3 - corrosive-injury/survival-time tissue study; no systematic HPE-utility outcome.
**Blanco Pampín et al. (2005)**	Gastric Mucosa Lesions in Drowning: Its Usefulness in Forensic Pathology	Category 3 - drowning gastric-mucosa diagnostic-marker study; not overall medicolegal HPE contribution/yield.
**Das et al. (2026)**	Post Mortem Findings and Histopathological Profile of Sudden Death: A Cross-sectional Study in a Tertiary Care Hospital, Tripura	Category 3 - descriptive sudden-death histopathology profile; no independent HPE-utility outcome.

Authors, year	Title	Primary exclusion reason
**Demirci et al. (2020)**	The Diagnostic Value of the Changes in the Renal Glomerulus in the Postmortem Diagnosis of Freshwater Drownings	Category 3 - drowning renal-glomerulus diagnostic-marker study; not routine HPE contribution/yield.
**Dorandeu et al. (2009)**	Value of Histological Study in the Fronto-Sphenoidal Suture for the Age Estimation at the Time of Death	Category 3 - forensic anthropology/age-estimation study; not an eligible HPE-utility outcome.
**Gauchotte et al. (2022)**	Myeloperoxydase and CD15 With Glycophorin C Double Staining in the Evaluation of Skin Wound Vitality in Forensic Practice	Category 3 - wound-vitality/IHC marker study; no autopsy HPE-contribution denominator.
**Goede et al. (2015)**	Wundalterdiagnostik nach letalem Schädel-Hirn-Trauma	Category 3 - biomarker/wound-age study; no eligible HPE-utility outcome or denominator.
**Hinnawi et al. (2019)**	Spectrum of Adrenal Changes - A Postmortem Study	Category 3 - adrenal lesion-spectrum study; no diagnostic-yield or HPE-contribution outcome.
**Kaur Jhajj et al. (2013)**	A Study of Histopathological Examination in Medico-Legal Autopsies in Faridkot, Punjab	Category 3 - descriptive histopathological spectrum study; no separate HPE contribution/change outcome.
**Manjula (2021)**	Spectrum of Histopathological Lesions in Lungs at Medico Legal Autopsy: A Ten Years Study	Category 3 - lung histopathology spectrum study; descriptive findings only, no HPE-utility outcome.
**Singh et al. (2023)**	The Role of Pathological Examination of the Liver in Medicolegal Autopsy: A Tertiary Care Center Study From North India	Category 3 - liver pathology profile study; no separate HPE contribution/change outcome.
**Suman et al. (2025)**	A Retrospective Study of Histopathological Changes in Liver in Medicolegal Autopsy: A Tertiary Care Center Study	Category 3 - liver histopathology frequency/profile study; no separate contribution/change outcome.
**Szleszkowski et al. (2015)**	Searching for New Features of Intravitality of Hanging Based on Macro- and Microscopic Evaluation of the Proximal Attachment of the Sternocleidomastoid Muscle and the Mastoid Process of the Temporal Bone	Category 3 – candidate-marker comparison in 35 known hanging deaths and 30 controls; reports lesion frequencies and gross–microscopic findings without an extractable contribution to a case-level death or vitality determination relative to a pre-HPE forensic assessment.
**Takahashi et al. (2022)**	Diagnostic Significance of the Histopathology of Bone Marrow Macrophages in Forensic Autopsies	Category 3 - bone marrow macrophage/agonal-condition marker study; no HPE-utility outcome.
**Tharakan and Wasnik (2023)**	Gross and Histopathological Study of Adrenal Glands from Routine Medico Legal Autopsies	Category 3 - adrenal gross/histology frequency study; no separate HPE-contribution outcome.

Authors, year	Title	Primary exclusion reason
**Todorović et al. (2011)**	Association of Pulmonary Histopathological Findings with Toxicological Findings in Forensic Autopsies of Illicit Drug Users	Category 3 - descriptive pulmonary histology/toxicology association study; no independent HPE utility/yield outcome.
**Yadav et al. (2019)**	An Autopsy Based Study of Adrenal Glands in Medicolegal Cases	Category 3 - adrenal pathology frequency study; no measured HPE contribution/change to COD.
**Ziyade et al. (2019)**	Evaluation of Salmonella Species Isolated in Post-Mortem Bacteriological Cultures in Autopsy Cases	Category 3 - postmortem microbiology study; histology not evaluated as a separable utility outcome.
**Byard et al. (2008)**	Diagnostic Yield and Characteristic Features in a Series of Decomposed Bodies Subject to Coronial Autopsy	Category 4 - decomposed-body diagnostic-yield study; HPE contribution not separately extractable.
**Cirielli et al. (2021)**	Consultation Between Forensic and Clinical Pathologists for Histopathology Examination After Forensic Autopsy	Category 4 – consultation and test-use audit combining gross re-examination, microscopy and other tests; no separately extractable HPE-contribution outcome with a defined pre-HPE baseline and denominator.
**Geller et al. (2022)**	Is It COVID-19? The Value of Medicolegal Autopsies During the First Year of the COVID-19 Pandemic	Category 4 - medicolegal COVID-19 autopsy study; microscopic findings not separable from other autopsy findings.
**Gupta et al. (2011)**	An Approach to Sudden Natural Deaths in Medicolegal Autopsies at Karamsad, Gujarat	Category 4 - sudden natural-death study; HPE contribution not separately extractable.
**Vural and Kalkan (2026)**	Decomposition and Medicolegal Autopsy in Geriatric In-Home Non-Traumatic Deaths: Histopathological Findings and a Public Health Perspective	Category 4 - integrated decomposed geriatric autopsy study; independent HPE contribution not separable.

Notes. *Categories correspond to the exclusion categories shown in Figure 1: Category 1, not an eligible original study (n = 5); Category 2, wrong population/setting (n = 2); Category 3, no HPE-utility outcome or denominator (n = 18); Category 4, histology effect not separately extractable (n = 5). Reports not retrieved: (1) Tabbara et al. (1962), "Apropos of the Value of Histological Examination in Medicolegal Autopsy (2nd Note)" (no DOI identified); (2) Subitha, K., and Rajeev, R. (2025), "Utility of Histopathological Examination in Medico Legal Autopsies," International Journal of Life Sciences Biotechnology and Pharma Research, 14(10), 685–688 (Embase accession L2042353702; DOI recorded in Embase: 10.69605/ijlbpr_14.10.2025.119). The Subitha report was present in the original Embase export, but its full text could not be retrieved during revision. The earlier design-based exclusion was unsupported and was removed; eligibility remains unresolved.*

Table S3. JBI critical appraisal of included studies: (A) item-level responses.

Study	JBI tool	Q1	Q2	Q3	Q4	Q5	Q6	Q7	Q8	Q9	Q10
Ambade 2016	JBI case series	Y	U	U	Y	Y	Y	U	Y	Y	Y
Bansal 2025	JBI case series	Y	U	U	Y	U	Y	Y	Y	Y	Y
Beer 2023	JBI case series	Y	Y	Y	U	Y	Y	Y	Y	Y	Y
Chaganti 2021	JBI case series	Y	U	U	Y	U	Y	Y	Y	Y	Y
Chatelain 2012	JBI case series	Y	Y	Y	Y	Y	Y	U	Y	Y	Y
Das 2025	JBI case series	Y	U	U	Y	U	Y	Y	U	Y	U
de la Grandmaison 2010	JBI case series	Y	Y	U	N	N	Y	Y	Y	Y	Y
Fronczek 2014	JBI case series	Y	Y	Y	Y	Y	Y	Y	Y	Y	Y
Goel 2023	JBI case series	U	U	U	U	U	Y	U	Y	Y	U
Hutchins 2012	JBI case series	Y	U	Y	Y	U	U	Y	Y	Y	Y
Karger 2004	JBI case series	Y	U	Y	Y	Y	Y	Y	Y	Y	Y
Langlois 2006	JBI case series	Y	Y	Y	Y	Y	Y	Y	Y	Y	Y
Matkowski 2021	JBI case series	Y	U	Y	Y	Y	Y	Y	Y	Y	Y
Molina 2007	JBI case series	Y	Y	Y	Y	Y	Y	Y	Y	Y	Y
Mukherjee 2025	JBI analytical cross-sectional	Y	Y	Y	Y	N	N	Y	Y	N/A	N/A
Perju-Dumbravă 2018	JBI case series	Y	U	Y	Y	U	U	Y	Y	Y	Y
Provenzano 2026	JBI case series	Y	Y	U	Y	U	Y	Y	Y	Y	Y
Rajakarunanaike 2025	JBI case series	Y	U	Y	Y	Y	Y	Y	Y	Y	Y
Shubhendu 2025	JBI case series	Y	U	Y	Y	Y	Y	Y	Y	Y	Y
Thomsen 2000	JBI case series	Y	Y	Y	Y	Y	Y	Y	Y	Y	Y
Tse 2012	JBI case series	Y	Y	Y	N	N	Y	Y	Y	Y	Y

Notes. *JBI, Joanna Briggs Institute; N, no; N/A, not applicable; U, unclear; Y, yes.*

For the JBI case-series checklist: *Q1, clear inclusion criteria; Q2, condition measured in a standard and reliable way; Q3, valid methods used to identify the condition; Q4, consecutive inclusion; Q5, complete inclusion; Q6, clear participant demographics; Q7, clear clinical information; Q8, outcomes or follow-up clearly reported; Q9, clear reporting of presenting site/clinic demographics; Q10, appropriate statistical analysis.*

For the JBI analytical cross-sectional checklist, used for Mukherjee et al.: *Q1, clear inclusion criteria; Q2, subjects and setting described; Q3, exposure measured validly and reliably; Q4, objective standard criteria used for the condition; Q5, confounding factors identified; Q6, strategies to deal with confounding stated; Q7, outcomes measured validly and reliably; Q8, appropriate statistical analysis; Q9–Q10, not applicable.*

Item responses are presented without summed scores or overall ratings. The appraisal procedure is described in Section 2.5 of the main manuscript; selection and extraction procedures are described separately in Sections 2.3 and 2.4. Panel B gives item rationales; Tables 1 and S5 describe other interpretive limitations.

Table S3 (continued). (B) Explanations of item-level responses.

Study	Explanation of checklist responses and relevant source caveat
Ambade 2016	Q2, Q3, and Q7 unclear: measurement reliability, condition-identification methods, and clinical information were insufficiently described.
Bansal 2025	Q2, Q3, and Q5 unclear: measurement and identification methods and complete inclusion were insufficiently described.
Beer 2023	Q4 unclear: consecutive inclusion was not clearly reported.
Chaganti 2021	Q2, Q3, and Q5 unclear: measurement and identification methods and complete inclusion were insufficiently described.
Chatelain 2012	Q7 unclear: clinical information was not fully reported.
Das 2025	Q2, Q3 and Q5 unclear: measurement and identification methods and complete inclusion were insufficiently described. Q8 and Q10 unclear: differing contribution labels and an unreconciled headline percentage prevent verification of clear diagnostic-outcome reporting and its numerical summary. The extractable technical limitation (2/28 complete autolysis) is retained; disputed diagnostic-contribution estimates are not synthesized.
de la Grandmaison 2010	Q3 unclear: standard histology was used, but the diagnostic criteria underlying the reported findings were not sufficiently specified to verify this item. Q4/Q5 no: the analyzed 428 cases were randomly sampled rather than consecutive or all eligible cases; the eligible sampling-frame size after exclusions was not given. Random sampling is not itself proof of biased selection. Other items: explicit exclusions, common organ protocol, two pathologists, aggregate demographics and forensic/clinical findings, site, and descriptive outcomes were reported.
Fronczek 2014	All applicable case-series items were rated Yes.
Goel 2023	Multiple items were unclear (Q1-Q5, Q7, and Q10), affecting confidence in inclusion, measurement, case ascertainment, and analysis.
Hutchins 2012	Q2, Q5, and Q6 unclear: measurement reliability, complete inclusion, and participant demographics were insufficiently described.
Karger 2004	Q2 unclear: standardized and reliable measurement of the condition was insufficiently described.
Langlois 2006	All applicable case-series items were rated Yes.
Matkowski 2021	Q2 unclear: standardized and reliable measurement of the condition was insufficiently described.
Molina 2007	All applicable case-series items were rated Yes.
Mukherjee 2025	Q5 and Q6 were rated No: confounding factors were not identified and strategies to address confounding were not reported.
Perju-Dumbravă 2018	Q2, Q5, and Q6 unclear: measurement reliability, complete inclusion, and participant demographics were insufficiently described.
Provenzano 2026	Q3 and Q5 unclear: condition-identification methods and complete inclusion were insufficiently described.
Rajakarunanaike 2025	Q2 unclear: standardized and reliable measurement of the condition was insufficiently described.
Shubhendu 2025	Q2 unclear: standardized and reliable measurement of the condition was insufficiently described.
Thomsen 2000	All applicable case-series items were rated Yes.
Tse 2012	Q4 and Q5 were rated No: 100 cases were randomly sampled, rather than included as a consecutive series or complete census of all eligible cases. Random sampling alone does not establish selection bias.

Table S4. Reporting limitations and proposed reporting elements for studies of HPE contribution.

Reporting element	Problem	Proposed reporting element	Example
Source population and case flow	Reporting only analyzed HPE cases without the total eligible autopsy source population.	Report eligible autopsies, HPE-submitted cases, available reports, analyzed cases, and exclusions.	Matkowski reported 141 HPE cases from 532 adult coronial autopsies; Provenzano required the flow from 1068 autopsies to 142 included cases.
Histology sampling strategy	Not stating whether HPE was routine, selective, organ-specific, mechanism-specific, referral-based, or retention-policy driven.	Describe the sampling rule, organs/tissues sampled, and whether selection was triggered by uncertainty or policy.	Selected-HPE studies were not interpreted as routine all-autopsy yield.
Pre-histology diagnostic baseline	Not defining what was known before microscopy.	State whether COD was gross-established, gross-pending, unascertained, or ancillary-pending before HPE.	Results Sections 3.3–3.5 distinguish source-described apparent, interim/mixed, and unresolved states. Broad cohorts are not assigned to an ordinal uncertainty tier.
Strict COD provision	Treating any diagnostic or significant microscopic finding as histology providing COD.	Count COD provision only when HPE established COD after gross autopsy did not provide one.	Langlois 49/203 and Matkowski 40/71 were treated as gross-pending/gross-unascertained COD-provision estimates.
Changed COD	Treating “added to,” “supported,” or “provided/altered” outcomes as changed COD.	Report changed COD only when HPE altered a gross or interim COD; otherwise retain the composite label.	Matkowski's Class I "provided or altered direct COD" (64) was decomposed using the study's own reporting into 40 provided (gross-unascertained) and 24 altered, so only the 24 are counted as changed COD; Fronczek's "added to COD" was treated as additive/supportive, not changed COD.
Manner or mechanism change	Combining COD change with manner, mechanism, or forensic interpretation.	Report COD change, manner change, and mechanism/forensic-interpretation change separately.	Beer’s single both-reviewer manner change was the same case as the COD change; Molina, Langlois, Tse, and Ambade reported no manner change.
Confirmation and refinement	Counting confirmation of gross findings as equivalent to independent COD provision or COD change.	Separate confirmation, refinement, additional pathology, and supportive information from strict COD outcomes.	Fronczek distinguished histology-alone COD provision from added information and confirmation; main Table 2 and Table S5 preserve these differences.
Composite outcomes	Reporting only broad categories such as “useful,” “contributory,” “diagnostic,” “conclusive,” or “provided/modified.”	Provide separate numerators and denominators for each component; if not possible, retain as composite.	Shubhendu's "provided or modified definitive COD" could not be split into clean provision/change components and was retained as a composite; Matkowski's "provided or altered direct COD," by contrast, was separable from the study's reported strata (40 provided + 24 altered).
Ancillary-test separability	Reporting final COD after histology, toxicology, microbiology, imaging, and scene review without separating HPE’s role.	State whether HPE was independently responsible, contributory with other tests, or non-separable.	Mukherjee’s HPE-based final opinions were treated cautiously because final opinion may incorporate HPE; toxicology-dominant contexts were not treated as pure HPE yield.

Reporting element	Problem	Proposed reporting element	Example
Vitality and injury timing	Pooling vitality, wound dating, or ligature-site interpretation with COD utility.	Report vitality/timing as a separate forensic-interpretation endpoint.	Chaganti and Perju-Dumbravă contribute targeted forensic interpretation, not general COD provision or a validated uncertainty–yield relationship.
Denominator-specific percentages	Calculating all percentages against a convenient denominator rather than the correct outcome denominator.	Report the numerator and denominator for every outcome; label all-autopsy, HPE-submitted, gross-pending, organ-specific, mechanism-specific, and special-subgroup denominators.	Matkowski 40/71 is the gross-unascertained estimate; 64/141 is a broader HPE-case composite and should not replace it.
Unreconciled contribution counts	Presenting an inconsistent or ambiguously defined source result as a stable yield estimate.	Describe the inconsistency, distinguish source records from synthesized outcomes, and omit the disputed numerical contribution from comparative summaries.	Das reports an extractable technical limitation (2/28 complete autolysis). Conflicting diagnostic-contribution categories are documented in Table S5; those estimates are excluded from main Table 2 and the numerical conclusions.
Non-contributory and limited histology	Combining nonspecific, irrelevant, autolyzed, technically inadequate, and missing histology.	Report no-impact, nonspecific, irrelevant, inconclusive, autolyzed, missing, and technically inadequate cases separately.	Ambade separated nonspecific findings from autolysis. Das excluded two completely autolyzed cases; that exclusion does not resolve the separate outcome-definition inconsistency.
Organ-specific cohorts	Applying heart-, brain-, lung-, or IHD-specific findings to all autopsies.	Label organ-specific scope and restrict interpretation to that organ/scenario.	Rajakarunanaike was interpreted as IHD/cardiac-context evidence rather than general all-autopsy yield.
Mechanism-specific or special-subgroup cohorts	Generalizing hanging, exhumation, alcohol-related, trauma, or custodial cohorts to routine practice.	State the target scenario and avoid extrapolation beyond that scenario.	Tse informs uncomplicated hanging; Karger informs exhumation/delayed-PMI context; Thomsen informs alcoholic medicolegal autopsy context.
Retention-policy studies	Treating organ-retention or sampling-policy outcomes as strict COD-yield estimates.	Distinguish whole-organ retention value, tissue-sampling sufficiency, forensic-conclusion contribution, and strict COD contribution.	Provenzano is retained as heart/brain retention and sampling-policy evidence; its broad utility endpoint is not a strict all-autopsy COD-change rate.
Mutual exclusivity	Not stating whether categories overlap.	State whether categories are mutually exclusive; if overlapping, provide rules for classification.	Main Table 2 and Table S5 identify study strata and composites so overlapping or alternative outcomes are not summed.
Interpretation language	Presenting selected or contextual findings as routine practice recommendations.	State what the evidence supports and what remains unknown; do not infer safe omission from a low frequency of changed diagnoses.	Main Table 3 sets out reporting implications and limits without ranking yield potential or recommending reduced sampling.

COD, cause of death; HPE, histopathological examination; IHD, ischemic heart disease; JBI, Joanna Briggs Institute; PMI, post-mortem interval. These reporting elements are review-derived suggestions, not a validated appraisal instrument or sampling guideline.

Table S5. Detailed denominator-specific outcomes and source caveats for all included studies.

Source-reported counts retain their original outcome definitions. They are not pooled. NR means not reported, not assessed, or not separately extractable; it is not zero. Multiple outcomes or strata from one study may overlap.

Study or stratum	Denominator and selection	Source-reported outcomes	Interpretive caveat
Tse et al. (2012) [32]	100 randomly selected uncomplicated hanging suicides; routine histology	COD change: 0/100. Manner or mechanism change: 0/100. Incidental findings only.	Uncomplicated hanging suicides with routine organ histology. Restricted mechanism comparator, not an omission-policy evaluation.
Molina et al. (2007) [26]	189 gross-clear routine medicolegal autopsies	COD change: 1/189. Manner change: 0/189. No COD/manner-relevant inheritable or communicable disease was identified.	Gross-uncertain, injury-dating, and disease-characterization cases were excluded by design.
Beer et al. (2023) [4]	200 selected trauma deaths (50 each: fire, immersion, suspension, traffic)	COD change: 1/200 by both reviewers; 8/200 by either reviewer. Manner change: 1/200 by both reviewers. Certainty increased in 17/192 cases without an either-reviewer COD change.	The 192 denominator excludes the 8/200 either-reviewer COD changes; the both-reviewer COD and manner change was the same drowning case.
Fronczek et al. (2014) [20]	287 HPE / 500 adult forensic cases	Histology-alone COD provision: 5/287. Gross findings confirmed: 61%; information added to COD: 8%; no COD influence: 30% (reported percentages among 287 HPE cases).	Broad selected HPE cohort, not a separately defined gross-clear stratum. Rounded source percentages are retained; exact event counts are not reconstructed from them. Added information is distinct from COD change.
Langlois (2006) [24] (interim-COD stratum)	435 interim-COD cases (of 638 adult coronial autopsies)	Interim COD modified: 21/435; confirmed: 158/435. Manner change: 0/638 across the full study.	Interim-COD stratum. Confirmation is supportive, not diagnostic change. The manner-change denominator of 638 covers the entire study and must not be counted again for the pending stratum.
Langlois (2006) [24] (pending-COD stratum)	203 pending gross-COD cases (of 638 adult coronial autopsies)	COD provision in gross-pending cases: 49/203. Manner change: 0/638 across the full study.	The 49/203 estimate was separately reported as COD provision in cases without a gross COD. The study's broader contributory category also included alteration and confirmation; 195/638 of the full cohort were non-contributory.
Ambade et al. (2016) [16]	263 HPE cases; 64 not-apparent subgroup; 2134 total medicolegal autopsies	COD provision in the not-apparent subgroup: 12/64. Manner change: 0/263. Gross COD confirmed: 70/263; specific findings: 117/263.	The broader 47/263 “helped give COD” includes 35 apparent-COD cases (support/refinement) and is not pure gross-pending provision; 138/263 nonspecific, 8/263 autolyzed.

Study or stratum	Denominator and selection	Source-reported outcomes	Interpretive caveat
Matkowski et al. (2021) [25]	141 HPE cases; 71 gross-unascertained subgroup	COD provision in gross-unascertained cases: 40/71. Interim COD altered: 24 cases within the 141-case HPE cohort. Indirect/part II contribution: 10/141; supportive/additional information: 52/141.	The 24 altered interim CODs fall within the 64/141 Class I direct-COD composite; Classes I–VI = 64 direct-COD, 10 indirect/part II, 52 supportive/additional, 15 irrelevant/inconclusive (total 141).
Mukherjee et al. (2025) [15]	87 convenience-sampled medicolegal autopsies; strata by gross status	HPE-based final opinion: 21/23 gross-pending; 8/16 pending with FSL investigation; 2/47 gross-established. These are source-defined final-opinion outcomes.	Composite HPE-based final opinion: 31/87 overall, comprising 2 gross-established, 21 gross-pending, 0 FSL-only, and 8 pending-with-FSL cases. The corresponding stratum denominators were 47, 23, 1, and 16. HPE contributed to the reference opinion. These are contextual final-opinion strata, not independently measured COD provision or change.
Shubhendu et al. (2025) [30]	172 analyzed HPE / 177 HPE-submitted / 262 medical-board autopsies	Definitive COD provided or modified: 33/172 (inseparable composite). Indirect contribution: 7/172; additional/supportive information: 131/172.	33/172 “definitive COD provided or modified” is a composite (provision vs change not separable; not entered in both columns); one case had no definitive COD correlation.
Bansal et al. (2025) [17]	54 HPE / 171 community brought-dead autopsies	COD moved from undetermined to a cardiac category: 3/54. Existing COD category changed: 4/54. Total source-attributed HPE COD effects: 7/54.	HPE effect was tabulated separately from toxicology; 47/54 HPE cases had no COD impact.
Chaganti et al. (2021) [18]	364 HPE submissions; 63 non-hanging/non-strangulation organ-HPE cases	Targeted ligature-site vitality assessment in hanging cases. Significant organ findings: 41/63 in the non-hanging/non-strangulation subgroup.	41/63 refers to the non-hanging/non-strangulation organ-HPE subgroup, not the 364-case cohort; not strict COD-yield evidence.
Perju-Dumbravă et al. (2018) [27]	16 neck-HPE cases / 66 hanging deaths	Neck HPE performed in 16/66 hanging autopsies. Hemorrhagic reaction: 11/16; compression/abrasion: 15/16. One case had microscopic findings supporting a gross suspicion of manual strangulation with subsequent suspension.	Small selected subgroup. Confirmation of an existing suspicion is a targeted forensic interpretation; no independently demonstrated new manner-of-death diagnosis is inferred.
Rajakarunanaike et al. (2025) [29]	100 deaths concluded as IHD; cardiac microscopic review	Microscopic findings establishing a COD not evident macroscopically: 9/100 (provision versus change not separable). Macroscopic/probable IHD confirmed: 57/100; alternatives excluded: 3/100.	Provision vs change not separable; 22/100 no findings; 9/100 positive but non-diagnostic (4 incompatible, 5 inconclusive); grades sum to 100; IHD-restricted, not general yield.

Study or stratum	Denominator and selection	Source-reported outcomes	Interpretive caveat
Hutchins et al. (2012) [22]	195 Miami-Dade brain-histology cases + 213 Mayo autopsies (complete neuropathology)	Mayo: brain histology provided COD in 7/213; contributed in 43% and confirmed in 74% (reported percentages, n = 213). Miami-Dade: cause-or-manner change, reported jointly, 0/184; pathology ruled out, 24/184.	Two cohorts with different institutional sampling. Miami-Dade reported cause-or-manner change jointly; no mechanism-change rate is inferred. Its denominator of 184 differs from the 195 cases with manner data. Mayo contribution and confirmation categories may overlap; rounded percentages are not converted into exact counts.
Chatelain et al. (2012) [12]	400 HPE / 536 forensic autopsies	Microscopic major diagnoses related to death: 35/400. Broad major diagnoses missed at autopsy: 83/400, comprising 35 microscopic diagnoses and 48 identified by additional gross examination. Confirmation or injury dating: 213/400. Non-contributory: 104/400.	The 400 HPE cases arose from 536 forensic autopsies. The 83/400 broad count is not microscopic yield. Minor unrelated findings were separate.
de la Grandmaison et al. (2010) [5]	428 randomly selected forensic autopsies from a source series of 1786; systematic organ HPE. Age 5–91 years; SIDS, skeletonized and organ-procurement cases excluded.	Histology-only COD provision: 36/428. Mechanism not shown grossly: 173/428. Manner affected: 56/428 (45 undetermined→natural, 8 accident→natural, 2 undetermined→homicide, 1 suspected suicide→natural). Prior medical information: 211/428. Injury documentation: 94/428. No significant addition: 59/428. Final COD unidentified: 32/428.	Broad unstratified cohort, not gross-clear or a defined unresolved stratum. Outcomes may overlap. The manner count is not wholly attributable to HPE alone: toxicology contributed in one case. Injury dating: 52/428; gunshot clarification: 35/428; traumatic nature ruled out: 6/428 and revealed: 1/428. Additional breakdowns and subgroup results are reported in the source article [5].
Karger et al. (2004) [23]	155 consecutive forensic exhumations	Histology described as decisive in 29% of the 103 cases in which COD was determined (rounded source percentage). Other reported utility included immunohistochemistry after long postmortem intervals.	Special delayed-PMI subgroup; histology and toxicology contributions partly combined; provision vs confirmation not cleanly separable.
Thomsen (2000) [31]	73 consecutive medicolegal autopsies in alcoholics	Histology described as decisive in 4/73 cases; COD changed in 1/73 (myocardial amyloid). Combined investigations helped distinguish natural from violent death in one DIC/Pneumococcus case. Toxicology was necessary in 37/73; microbiology was supportive.	Restricted alcoholic-death cohort with histology, toxicology, and microbiology interpreted together. Treated as special-context evidence rather than strict pre-/post-HPE manner-change evidence; do not generalize to routine all-autopsy yield.
Goel et al. (2023) [21]	100 selected HPE medicolegal cases	Author-defined “conclusive” findings: 41/100. Provision versus change was not separable. Gross–microscopic discrepancies were also described.	Not used for strict COD-yield synthesis: the total-autopsy denominator and the operational definition of “conclusive” are unclear.

Study or stratum	Denominator and selection	Source-reported outcomes	Interpretive caveat
Provenzano et al. (2026) [28]	142 analyzed / 158 reports / 225 HPE ordered / 1068 autopsies; organ: heart 138, brain 135	Author-defined diagnostic findings: 22/142 (heart 13/138; brain 2/135). Discordant findings: 4/142 (heart 1; brain 2). Manner clarified in 13/58 undetermined cases; 2 cases reclassified to undetermined. Confirmatory findings: 48/142; prior-condition information: 14/142. Organ-useful findings: heart 60/138; brain 19/135.	Strict COD provision not separately extractable; injury characterization 29/142; non-contributory 21/142; minor unrelated 4/142; broad useful endpoint, not strict all-autopsy yield; retention/sampling-policy context.
Das et al. (2025) [19]	28 selected HPE cases; 2 completely autolyzed; 26 analyzable (14 obscure-COD, 12 diseased-organ)	Technically limited HPE: complete autolysis in 2/28, excluded from the source diagnostic-contribution analysis. Among 26 analyzable cases, source Table 3 lists six obscure cases with specific organ-related findings, eight with comorbidity, and 12 diseased-organ cases with sufficient findings. Source Table 4 records 18 positive entries (12 + 6) under a comorbid-pathology heading; the discussion reports 76.92% sufficient to explain COD.	The technical limitation is extractable and falls within the eligible outcomes. The diagnostic-contribution figures remain unreconciled: 18/26 is 69.23%, whereas 76.92% corresponds to 20/26, with differing endpoint labels. These source entries are documented for transparency; no numerical COD-provision, change, or overall-yield estimate is synthesized.

Study citations correspond to the reference list in the main manuscript. HPE, histopathological examination; COD, cause of death; FSL, forensic science laboratory; IHD, ischemic heart disease; CNS, central nervous system; PMI, post-mortem interval.
